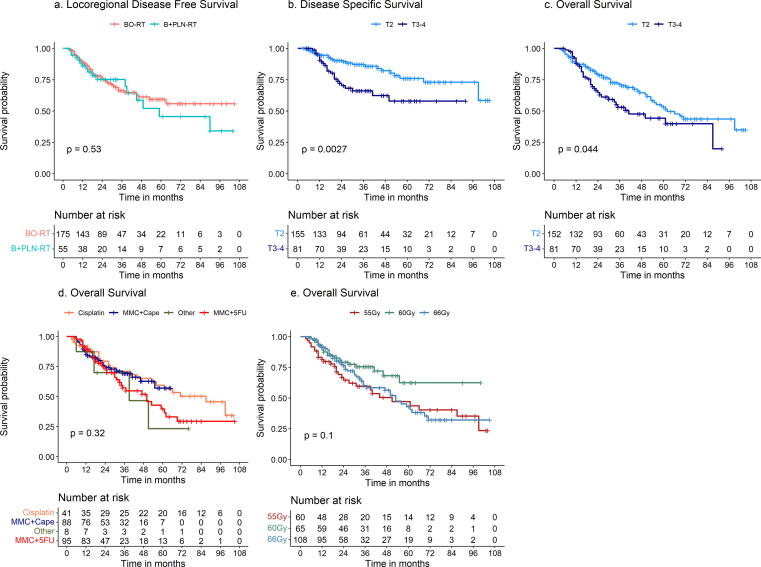# A Multicenter Retrospective Cohort Series of Muscle-invasive Bladder Cancer Patients Treated with Definitive Concurrent Chemoradiotherapy in Daily Practice

**DOI:** 10.1016/j.euros.2022.02.010

**Published:** 2022-03-16

**Authors:** Ben-Max de Ruiter, Maaike W. van de Kamp, Jonah P.Z. van Steenbergen, Martine Franckena, Joost L. Boormans, Jeantine M. de Feijter, Adriaan D. Bins, Maarten C.C.M. Hulshof, Theo M. de Reijke, Eva Schaake, Jorg R. Oddens

**Affiliations:** a Department of Urology, Amsterdam University Medical Centers, University of Amsterdam, Amsterdam, The Netherlands; b Department of Urology, Netherlands Cancer Institute – Antoni van Leeuwenhoek Hospital, Amsterdam, The Netherlands; c Department of Radiotherapy, Erasmus MC Cancer Institute, Erasmus University Medical Center, Rotterdam, The Netherlands; d Department of Urology, Erasmus MC Cancer Institute, Erasmus University Medical Center, Rotterdam, The Netherlands; e Department of Medical Oncology, Netherlands Cancer Institute – Antoni van Leeuwenhoek Hospital, Amsterdam, The Netherlands; f Department of Medical Oncology, Amsterdam University Medical Centers, University of Amsterdam, Amsterdam, The Netherlands; g Department of Radiation Oncology, Amsterdam University Medical Centers, University of Amsterdam, Amsterdam, The Netherlands; h Department of Radiation Oncology, Netherlands Cancer Institute – Antoni van Leeuwenhoek Hospital, Amsterdam, The Netherlands

**Keywords:** Carcinoma, Transitional cell, Chemoradiotherapy, Therapeutics

## Abstract

**Background:**

Concurrent chemoradiotherapy (CRT) as a definitive treatment option for patients with nonmetastatic muscle-invasive bladder carcinoma (MIBC) is increasingly being applied in clinical practice.

**Objective:**

To assess the oncological and toxicity outcomes in a contemporary cohort of nonmetastatic MIBC patients treated with concurrent CRT in daily practice.

**Design, setting, and participants:**

Patients with nonmetastatic MIBC (cT2-4aN0M0) who had received CRT with curative intent between January 2010 and April 2020 in three centers were retrospectively identified. The CRT consisted of 66 Gy (or biologically equivalent) plus either mitomycin C and fluorouracil/capecitabine or cisplatinum.

**Outcome measurements and statistical analysis:**

The primary endpoint was the 2-yr locoregional disease-free survival (LDFS) estimate. Secondary endpoints were complete response, disease-specific survival (DSS), overall survival (OS), bladder intact event-free survival (BI-EFS), and severe adverse events (<90 d of starting CRT). Kaplan-Meier survival and Cox multivariable regression analyses were performed.

**Results and limitations:**

We included data of 240 MIBC patients with a median age of 74 yr and a median follow-up of 27 mo (interquartile range 11–44). Complete response on first cystoscopy after CRT was seen in 209 cases (90%). The 2-yr LDFS was 76% (95% confidence interval [CI] 70–82%); the 5-yr OS and DSS were 50% (95% CI 42–59%) and 70% (95% CI 62–79%), respectively. On multivariable analysis, cT2 versus cT3–4 tumor stage was significantly associated with better DSS (hazard ratio 1.02, 95% CI 1–1.05, *p* = 0.024). The 2-yr BI-EFS was 75% (95% CI 69–82%). Forty-three (17%) patients experienced a severe adverse event (grade ≥3). Limitations include retrospective design and heterogeneous administration of CRT.

**Conclusions:**

Concurrent CRT is a safe and effective treatment modality for nonmetastatic MIBC.

**Patient summary:**

Chemoradiotherapy for the treatment of muscle-invasive bladder carcinoma is increasingly being applied. In this study, we reviewed the outcomes of this bladder-sparing treatment using a series of patients treated in three hospitals in daily practice. We found that administration of chemoradiotherapy can be safe and effective.

## Take Home Message

Chemoradiotherapy is a safe and effective treatment option for a wide range of muscle-invasive bladder carcinoma patients with 2-yr locoregional disease-free-survival of 76%. Results in daily practice are comparable with trial outcomes. Lymph node irradiation and carcinoma in situ were not related to outcomes.

## 1 Introduction

Bladder cancer (BC) is among the ten most frequently diagnosed types of cancers for men and women in the Netherlands, with over 6000 new cases in 2019 [1]. A key distinction is made between non–muscle-invasive bladder cancer (NMIBC) versus muscle-invasive bladder cancer (MIBC). While NMIBC can be treated with transurethral resection of a bladder tumor (TURBT) followed by intravesical instillations alone, MIBC requires additional treatment.

Despite treatment, an estimated 50% of patients with nonmetastatic MIBC are alive 5 yr after diagnosis. Treatment guidelines advocate radical cystectomy (RC) plus lymph node dissection ± neoadjuvant chemotherapy (NACT) as a primary treatment option for MIBC, with chemoradiotherapy (CRT) as an alternative for patients who wish to preserve the bladder or for patients not fit for surgery [1]. In 2019, about half of MIBC patients in the Netherlands received RC, with or without NACT and about a quarter received radiotherapy (RT), with or without concurrent chemotherapy (CTx) [2]. Since the BC2001 trial (published in 2012) confirmed the superiority of RT with concurrent CTx for MIBC over RT alone, the focus is slowly shifting to bladder-sparing treatment (BST) through trimodality therapy (TMT) as an alternative for RC [3]. TMT includes maximal TURBT followed by RT and concurrent *radiosensitizing* CTx. The long-term oncological results in series from large centers and reported in systematic reviews were comparable between RC and TMT [4], [5], [6], [7]. Currently, TMT is offered to well-informed selected patients who opt for BST or for whom RC is not a feasible option.

The aim of this study is to establish oncological and toxicity outcomes in a contemporary series of patients treated with CRT for localized nonmetastatic MIBC in three large centers in the Netherlands.

## 2 Patients and methods

### 2.1 Patients

This retrospective cohort study was approved by the Institutional Review Board of the Academic Medical Center Amsterdam, the Netherlands (IRB W20_416#20.463). Patients with nonmetastatic MIBC (cT2–4 N0), who had received CRT between January 2010 and April 2020 in Academic Medical Center Amsterdam, Erasmus University Medical Center Rotterdam, or the Netherlands Cancer Institute, were retrospectively identified. We analyzed data of patients with suspected pelvic lymph node (PLN) metastasis on computed tomography (CT) who underwent a pelvic lymph node dissection (PLND) before CRT and had pathological tumor-negative lymph nodes. Additional eligibility criteria were over 18 yr of age and having received CTx concurrent to RT. Patients with second malignancies or predominantly nonurothelial BC (<50% urothelial carcinoma) were excluded from analyses, as were those treated with concurrent immunotherapy or palliative intent.

### 2.2 Pretreatment staging and NACT

The three participating hospitals adhered to the European guidelines for MIBC [1]. Patients underwent physical examination, cystoscopy, and CT scan of the chest and abdomen as pretreatment staging. T stage was determined based on CT. The decision to apply TMT was made by a multidisciplinary team.

NACT was generally offered to patients with high-risk features, such as T3-T4a disease, suspicion of PLN metastasis on radiology, or histopathological risk factors on TURBT, such as lymphovascular invasion or variant histology.

### 2.3 Chemoradiotherapy

CRT was administered using volumetric modulated arc therapy or intensity-modulated radiotherapy (IMRT). Tumors were irradiated with doses equivalent to 64–66 Gy in 2 Gy fractions in a continuous course: 55 Gy (20 × 2.75 Gy) in 4 wk, 60 Gy (25 × 2.4 Gy) in 5 wk, or 66 Gy (33 × 2 Gy) in 6.5 wk, depending on local hospital guidelines. One of the three hospitals had applied elective PLN irradiation. PLNs were irradiated along the internal iliac artery until the level of the common iliac artery. Solitary tumors were demarcated using lipiodol injections for simultaneous integrated boost [8]. Multifocal BC was treated with whole bladder irradiation in all hospitals.

**Fig. 1:**
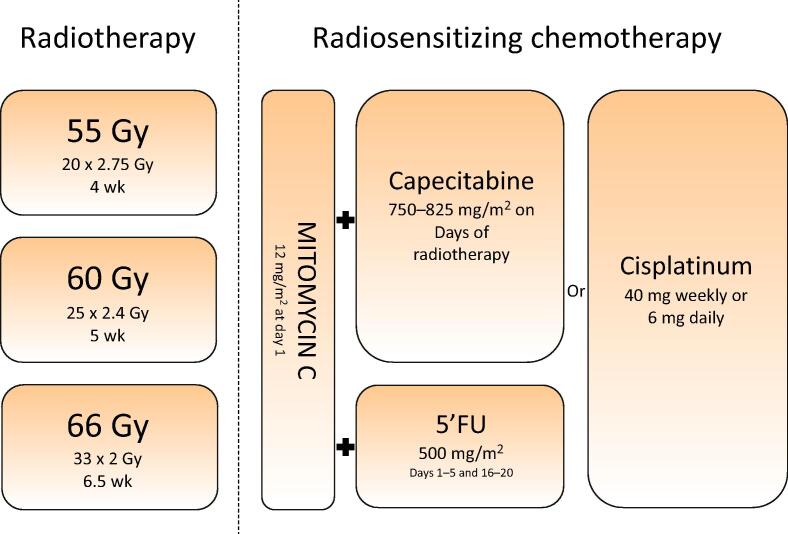
Chemoradiotherapy combinations. Gy = Gray; 5′FU = fluorouracil.

Three different regimens of concurrent CTx were applied: mitomycin C (MMC) + fluorouracil, MMC + daily capecitabine, or low-dose cisplatin (Fig. 1).

### 2.4 Follow-up

Follow-up consisted of serial cystoscopy in combination with urine cytology and CT scans. The first cystoscopy was generally performed 3 mo after the start of TMT. None of the three hospitals performed standard tumor site biopsies following CRT.

### 2.5 Outcomes

The primary endpoint of the study was locoregional disease-free survival (LDFS) at 2 yr of follow-up, defined as survival free of recurrence in pelvic nodes or bladder (data censored at first sign of local recurrence, metastasis, or death). Oncological and toxicity outcomes were obtained by chart review. Oncological outcomes were measured from the date of diagnosis to the date of the first documented event. Disease-specific survival (DSS) was defined as surviving treatment with the cause of death not related to BC (data censored at death of other cause than BC). Bladder intact event-free survival (BI-EFS) was defined as the first occurrence of any of the following events: (1) residual/recurrent MIBC (confirmed by TURBT), (2) nodal or distant metastases as assessed by CT and/or biopsy results, (3) salvage cystectomy (SC), and (4) death. BI-EFS has been proposed to be a clinically relevant composite outcome measure to assess bladder preservation and oncological safety of BST [9]. Acute toxicity (<90 d of starting CRT) was retrospectively assigned according to the Common Terminology Criteria for Adverse Events (CTCAE) v5.0 [10]. Hematological toxicities were evaluated for the duration of treatment only.

### 2.6 Statistical analysis

Baseline and treatment characteristics, as well as toxicity details, are reported using descriptive statistics. Survival outcomes were estimated using the Kaplan-Meier method. A univariate analysis on preselected covariates (Supplementary Table 1) was performed to select covariates for the multivariable regression analysis. For a stepwise multivariable regression analysis, we used the Cox model. The proportionality assumption was tested with the use of Schoenfeld residuals. All statistical analyses were two sided, and a *p* value of <0.05 was considered statistically significant. The Kaplan-Meier survival analysis was conducted in R version 4.03 (R Foundation for Statistical Computing, Vienna, Austria). Other analyses were performed using IBM SPSS Statistics version 26.0 (IBM Corp., Armonk, NY, USA).

## 3 Results

### 3.1 Patient selection

We identified 286 patients treated with TMT. After exclusion of nonurothelial carcinoma (*n* = 11), T1 disease (*n* = 4), pN1 or cN1 without negative PLND (*n* = 20), unresectable disease (*n* = 3), and sequential NACT followed by RT alone (*n* = 8), data of 240 patients were eligible for analysis.

### 3.2 Baseline and treatment characteristics

Baseline patient and treatment characteristics are displayed in Table 1, Table 2. The median follow-up was 27 mo (interquartile range [IQR] 11–44). Gemcitabine + cisplatin (*n* = 23) was the most used form of CTx if NACT (*n* = 31) was administered. Tumor regression on CT scan after NACT was seen in 26 (84%) cases. Elective PLN irradiation was applied in 24% (*n* = 57) of cases.

**Table 1:** Baseline characteristics

Age (yr), median (IQR)		74 (67–81)	
		*n*	%
Total number of patients		240	100
Sex	Male	187	78
	Female	53	22
WHO	0	132	56
	1	97	41
	2	8	3
	NR	3	1
CCI	0–2	35	15
	3–5	138	58
	>5	63	27
	NR	4	2
T stage	T2	159	66
	T3	67	28
	T4a	14	6
N stage	0	237	98
	1	6	3
Hydronephrosis at the start of CRT	Yes	38	17
TURBT histology	100% urothelial	203	85
	Urothelial + squamous	17	7
	Urothelial + sarcomatoid	8	3
	Urothelial + micropapillary	5	2
	Urothelial + small cell	1	0
	Urothelial + glandular	6	3
Tumor location	Dome	15	6
	Lateral	96	40
	Trigone and up	51	21
	Anterior	14	6
	Posterior	23	10
	Diverticulum	4	2
	Multifocal	35	15
	NR	2	1
Concomitant CIS	Yes	48	20
Radical TURBT	Yes	152	63
	No	77	32
	Not reported	11	5
Size of tumor (cm)	<3	45	19
	>3	95	40
	Not reported	100	42
Elective lymph node irradiation	Yes	57	24
	No	184	76

CCI = Charlson comorbidity index; CIS = carcinoma in situ; CRT = chemoradiotherapy; IQR = interquartile range; NR = not recorded; TURBT = transurethral resection of a bladder tumor; WHO = World Health Organization performance status.

**Table 2:** Treatment characteristics

Time TURBT – start CRT (d), median (IQR)	CRT CRT + NACT	63 (57–76) 181 (151–211)	
		*n*	%
NACT	Yes	31	13
Radiotherapy schedule	20 × 2.75 Gy	61	25
	25 × 2.4 Gy	65	27
	33 × 2 Gy	114	48
Radiosensitizing CTx	MMC + Cape	88	37
	MMC + 5′FU	101	42
	Cisplatin	43	18
	Other	8	3
Dose reduction CTx	Yes	36	15
PLND	Yes	19	8
Salvage cystectomy	Yes	9	4

Cape = capecitabine; CRT = chemoradiotherapy; CTx = chemotherapy; 5′FU = fluorouracil; IQR = interquartile range; MMC = mitomycin C; NACT = neoadjuvant chemotherapy; PLND = pelvic lymph node dissection; TURBT = transurethral resection of a bladder tumor.

### 3.3 Oncological outcomes

#### 3.3.1 Response on first cystoscopy after CRT and LDFS

The median time to first reported cystoscopy was 4 mo (IQR 3–6). In eight cases (3%), data on first cystoscopy was missing. A complete response on first cystoscopy after CRT was seen in 209 of the remaining 232 patients (90%). For 15 patients (7%), residual tumor was suspected, for which either TURBT or follow-up cystoscopy was performed, depending on performance status. In eight cases (3%), no response was observed.

**Fig. 2:**
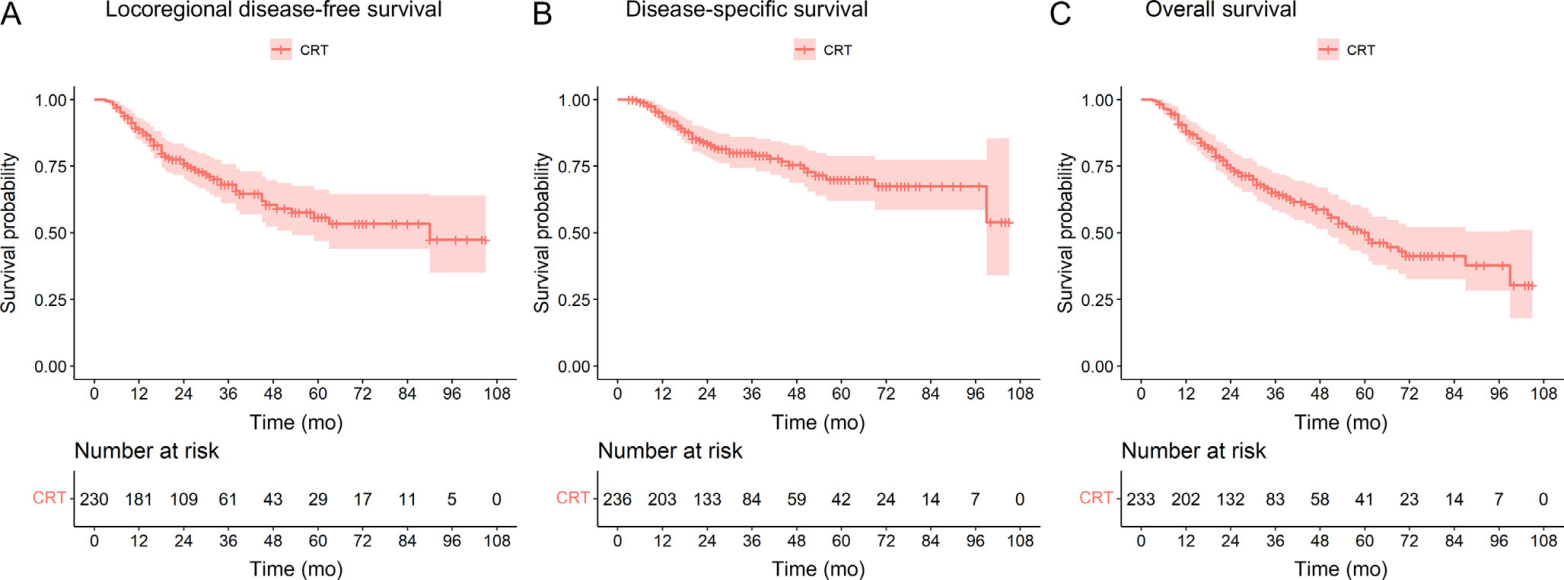
Kaplan-Meier survival analysis showing survival probabilities: (A) locoregional disease-free survival, (B) disease-specific survival, and (C) overall survival. CRT = chemoradiotherapy.

LDFS findings are displayed in Figure 2A, and in Supplementary Figure 2A comparing CRT of the bladder alone to CRT of the bladder in combination with elective PLN. LDFS probabilities for 1, 2, and 5 yr after diagnosis were 0.89 (95% confidence interval [CI] 0.85–0.93), 0.76 (95% CI 0.70–0.82), and 0.56 (95% CI 0.47–0.66), respectively. The 5-yr local recurrence rates for NMIBC, MIBC, and regional lymph node metastasis after CRT were 11%, 7%, and 8%, respectively.

#### 3.3.2 Disease-specific survival

DSS findings are displayed in Figure 2B and Supplementary Figure 2B. The DSS probabilities for 1, 2, and 5 yr after diagnosis are 0.94 (95% CI 0.91–0.97), 0.83 (95% CI 0.0.78–0.89), and 0.70 (95% CI 0.62–0.79), respectively. The most common sites of progression were multifocal sites (38%), bone metastases (18%), and retroperitoneal metastases (15%), followed by lung (9%) and peritoneal metastases (6%).

#### 3.3.3 Overall survival

OS findings are displayed in Figure 2C. The OS probabilities for 1, 2, and 5 yr after diagnosis are 0.88 (95% CI 0.84–0.93), 0.74 (95% CI 0.69–0.81), and 0.50 (95% 0.42–0.59), respectively. OS significantly differed between patients with T2 versus T3–4 tumors (Supplementary Fig. 2C). No association was found for RT dosage or CTx radiosensitizer (Supplementary Fig. 2D and 2E). NACT was not a predictor of OS (*p* = 0.55).

### 3.4 Multivariable analyses

Clinical T stage, presence of hydronephrosis at the start of CRT, and radicality of the TURBT were significantly associated with OS and DSS in the univariable analysis (Supplementary Table 1). None of the baseline or treatment parameters, including carcinoma in situ (CIS), hydronephrosis, and elective PLN irradiation, were significantly associated with LDFS. In a multivariable analysis for OS, none of these covariates were significantly associated with OS. In a multivariable analysis for DSS, T3–4 tumors were significantly associated with a higher risk of disease-specific mortality. Furthermore, hydronephrosis lost significance, although a trend for worse OS and DSS was apparent (hazard ratios [HRs] of 1.8 and 1.7, respectively). Results of the multivariable analyses are shown in Table 3.

**Table 3:** – Cox multivariable regression analysis

Covariates	Comparison	OS			DSS		
		HR	95% CI	*p* value	HR	95% CI	*p* value
Clinical T stage	T2 vs T3–4	1.01	0.99–1.02	0.469	1.02	1–1.05	0.024
Radical TURBT	Radical vs not radical	0.71	0.45–1.12	0.141	0.82	0.44–1.52	0.534
Baseline hydronephrosis	Present vs not present	1.80	1.00–3.25	0.052	1.70	0.80–3.60	0.166

CI = confidence interval; DSS = disease-specific survival; HR = hazard ratio; OS = overall survival; TURBT = transurethral resection of a bladder tumor.

### 3.5 Bladder intact event-free survival

**Supplementary figure 1:**
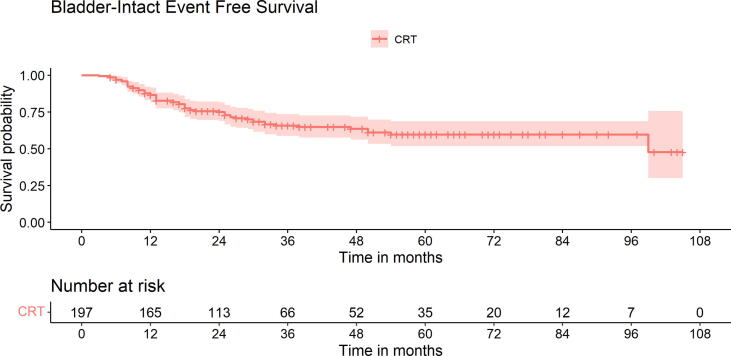


BI-EFS findings are displayed in Supplementary Figure 1. BI-EFS probabilities for 1, 2, and 5 yr after diagnosis are 0.87 (95% CI 0.82–0.92), 0.75 (95% CI 0.69–0.82), and 0.60 (95% CI 0.52–0.69), respectively. Fourteen patients (7%) had MIBC recurrences on TURBT. Nine of them underwent SC, three were unfit for salvage surgery, one opted for palliative RT, and another had concomitant pulmonary metastasis and received palliative CTx. Pathology results after SC revealed one pT4b tumor, one pT4a tumor, three pT3a tumors, and two cases of CIS. In two patients, no residual disease was found.

### 3.6 Toxicity and bladder symptoms

Table 4 displays data on toxicity outcomes within 90 d of starting CRT.

**Table 4:** Acute toxicity (<90 d) scored according to the CTCAE v5.0

Adverse event a	Grade <3		Grade 3		Grade 4	
	*n*	%	*n*	%	*n*	%
Highest scored	149	61	33	14	8	3
Genitourinary	101	42	23	10	4	2
Frequency	43	18	1	0	0	0
Cystitis, noninfective	46	19	4	2	1	0
Urinary tract infection	7	3	7	3	3	1
Urinary tract obstruction	0	0	3	1	0	0
Urinary retention	5	2	6	3	0	0
Hematuria	3	1	2	1	0	0
Gastrointestinal	44	18	2	1	3	1
Diarrhea	38	16	2	1	0	0
Fistula	0	0	2	1	2	1
Nausea	3	1	0	0	1	0
Obstipation	6	3	1	0	0	0
Hematologi cal	35	14	6	3	1	0
Thrombocytopenia	29	12	3	1	1	0
Thromboembolic event	0	0	3	1	0	0
Miscellaneous	6	3	0	0	0	0
Other	36	15	3	1	0	0
Fatigue	26	11	0	0	0	0
Mucositis	1	0	1	0	0	0
Pneumonia	0	0	2	1	0	0
Miscellaneous	9	4	0	0	0	0

AE = adverse event; CTCAE = Common Terminology Criteria for Adverse Events.

a Patients could experience more than one AE.

## 4 Discussion

The aim of the current study was to present an overview of oncological and toxicity outcomes in a contemporary cohort series of patients treated in daily practice with TMT for MIBC in three larger centers in the Netherlands. We report a 2-yr LDFS rate of 76% and 5-yr OS and DSS rates of 50% and 70%, respectively. A higher T stage was negatively correlated with disease-free survival. Of the patients, 7% developed a muscle-invasive recurrence in the bladder. Severe adverse events (grade ≥3) within 90 d of starting CRT occurred in 17% of patients. BI-EFS at 2-yr follow-up was 75%.

The BC2001 trial (2012) was a landmark randomized controlled trial for RT alone versus TMT [3]. In the TMT group, the 2-yr LDFS rate was 67% and the 5-yr OS rate was 48%; these outcomes were superior to those of RT alone. Giacalone et al [4] published the largest single-center retrospective series to date, including 475 patients treated from 1986 to 2013 in the Massachusetts General Hospital, over a variety of clinical trials, and reported 5-yr OS and DSS of 57% and 66%, respectively. Mak et al [11] presented a retrospective pooled analysis of six prospective Radiation Therapy Oncology Group studies, and reported 5-yr OS and DSS of 57% and 71%, respectively. The present study shows comparable outcomes. A key distinction between these three studies and our results is performing tumor-site biopsies routinely, which was not a standard procedure in the current study. The results of the current study suggest that this omission does not compromise oncological safety and adds to the feasibility of TMT. In addition, the present study reflects daily practice, including patients who might not have been suitable for the reported clinical studies due to comorbidity and frailty.

Interestingly, only clinical tumor stage proved to be significantly associated with DSS on the multivariable analysis with an HR of 1.02. In previous studies, T3–4 stage and tumor-associated CIS were negatively correlated with survival data [4], [12]. Although in the present study, both the presence of hydronephrosis and incomplete TURBT in the univariate analysis were associated with outcome data, this was not the case for tumor-associated CIS—perhaps because patients with predominantly CIS might have been counseled for RC. Nevertheless, our results suggest that patients, including those with limited CIS in combination with MIBC, can effectively be treated with bladder-sparing TMT.

Another debated topic related to CRT treatment is the desirability of elective lymph node irradiation [13]. In a recent consensus meeting of the European Association of Urology and European Society of Medical Oncology, a majority of stakeholders preferred to electively irradiate PLNs in case of CRT [14]. A clinical trial published in 2016, comparing whole-pelvis irradiation with bladder-only irradiation, did not find an improved local control rate with whole pelvis irradiation, although this was associated with higher toxicity than irradiating the bladder only [15]. A confounding factor in whole-pelvis irradiation without elective node target volume is that the generally applied margins of 1–2 cm around the bladder will include the closest lymph nodes. In the present study, the addition of elective PLN irradiation did not impact local control rate or survival over whole bladder only.

Moreover, we found a mild toxicity profile of CRT, favorable to earlier results [3], [5]. This could be a result of recent advances in RT techniques, such as IMRT and the use of a simultaneous bladder boost, which reduce radiation on surrounding tissue [16]. Furthermore, the use of capecitabine avoids the need for hospital admission and infusion pumps, adding to the feasibility of CRT [9], [17].

Despite the proven safety and feasibility of TMT for MIBC, it is still not widely accepted in clinical practice. Early disappointing results of RT only for BC might have contributed to clinicians’ negative opinion of RT-based therapies for BC, so that they consequently reserve RT mostly for frail patients unfit for surgery [18], [19]. The British SPARE trial, which did not meet recruitment target, has proved that a randomized comparison between BST and RC is not feasible due to patients’ and clinicians’ preferences [20]. Several systematic reviews have indicated that current literature is biased and provides conflicting results, confirming the lack of an adequate comparison and, therefore, possible slow acceptance [7], [21].

Recent advancements with checkpoint inhibitors (CPIs) might accelerate the application of BST. Early results have shown remarkable results of CPIs in combination with RC [22], [23], [24]. Although no data on the combination of CPIs and TMT are available, several ongoing trials are testing CPIs only and combination of CPIs with CRT, and results are to be expected in the near future [25].

Strengths include the large number of patients, stringent selection criteria, inclusion of cN0 patients and those with pathological tumor-negative lymph nodes, and availability of oncological and toxicity results, thereby providing insight into daily practice of TMT. Limitations are the retrospective nature of the study with its potential underscoring of acute toxicity and the absence of nongenitourinary late toxicity scoring.

To our knowledge, we present the second largest multicenter retrospective cohort of MIBC patients treated with CRT. This study is an extension of the single-center cohort published by Voskuilen et al [9].

## 5 Conclusions

Our primary findings reproduce LDFS, DSS, and OS rates in daily practice that are comparable with those of earlier published series, with a low rate of SC performed [4]. Furthermore, this study confirms in the multivariate analysis that patients with T2 versus T3–4 tumors treated with CRT are at a slightly lower risk of disease-specific mortality. In addition, the univariate and multivariate analyses suggest that patients with limited CIS in combination with MIBC can be considered for CRT, and that the addition of elective PLN irradiation does not improve the local control rate or survival outcomes compared with whole bladder only. Moreover, our results provide evidence of safe CRT treatment strategies, with a favorable acute toxicity profile.

  ***Author contributions*:** Ben-Max de Ruiter had full access to all the data in the study and takes responsibility for the integrity of the data and the accuracy of the data analysis.

*Study concept and design*: de Ruiter, van de Kamp, van Steenbergen, Franckena, Boormans, Bins, Hulshof, de Reijke, Schaake, Oddens.

*Acquisition of data*: de Ruiter, van de Kamp, van Steenbergen, Franckena, Boormans, Schaake.

*Analysis and interpretation of data*: de Ruiter, van de Kamp, van Steenbergen, Schaake, Oddens.

*Drafting of the manuscript*: de Ruiter, van de Kamp, van Steenbergen.

*Critical revision of the manuscript for important intellectual content*: de Ruiter, van de Kamp, van Steenbergen, Franckena, Boormans, de Feijter, Bins, Hulshof, de Reijke, Schaake, Oddens.

*Statistical analysis*: de Ruiter, van Steenbergen, van de Kamp.

*Obtaining funding*: de Reijke, Oddens.

*Administrative, technical, or material support*: Franckena, Boormans, Bins, Hulshof, de Feijter, de Reijke, Schaake, Oddens.

*Supervision*: Franckena, Boormans, Bins, Hulshof, de Reijke, Schaake, Oddens.

*Other*: None.

  ***Financial disclosures:*** Ben-Max de Ruiter certifies that all conflicts of interest, including specific financial interests and relationships and affiliations relevant to the subject matter or materials discussed in the manuscript (eg, employment/affiliation, grants or funding, consultancies, honoraria, stock ownership or options, expert testimony, royalties, or patents filed, received, or pending), are the following: None.

  ***Funding/Support and role of the sponsor*:** The work of Ben-Max de Ruiter is funded by the Cure for Cancer foundation and the Dutch Cancer Society.

## Figures and Tables

**Supplementary figure 2:**