# Supplementary material for: A Multicenter Retrospective Cohort Series of Muscle-invasive Bladder Cancer Patients Treated with Definitive Concurrent Chemoradiotherapy in Daily Practice
Source: Eur Urol Open Sci. 2022 Mar 16;39:7–13. doi: 10.1016/j.euros.2022.02.010 (PMC9068732; doi:10.1016/j.euros.2022.02.010)
Supplement: Supplementary data 1 [file mmc1.docx]

| **Supplementary Table 1: Univariate regression analysis** | | | | | | | | | | |
| --- | --- | --- | --- | --- | --- | --- | --- | --- | --- | --- |
|  |  | **OS** | | | **DSS** | | | **LDFS** | | |
| **Covariates** | **Comparison** | **HR** | **95%CI** | **p** | **HR** | **95%CI** | **p** | **HR** | **95%CI** | **p** |
| **Clinical T stage** | T2 vs T3-4 | 1.01 | 1.00-1.02 | **0.047** | 1.03 | 1-1.05 | **0.004** | 1.01 | 0.99-1.03 | 0.188 |
| **Radical TURBT** | Radical vs irradical | 0.62 | 0.41-0.95 | **0.028** | 0.71 | 0.39-1.27 | 0.249 | 1.05 | 0.56-1.98 | 0.872 |
| **Baseline hydronephrosis** | Not present vs present | 2.12 | 1.25-3.60 | **0.005** | 2.67 | 1.37-5.20 | **0.004** | 1.35 | 0.60-3.05 | 0.464 |
| Concomitant CIS | No vs yes | 1.08 | 0.64-1.81 | 0.781 | 1.16 | 0.58-2.34 | 0.673 | 1.08 | 0.54-2.17 | 0.832 |
| Size of the tumor | <3cm vs >3cm | 0.99 | 0.94-1.05 | 0.810 | 0.99 | 0.92-1.06 | 0.784 | 1.00 | 0.94-1.08 | 0.934 |
| Multifocal tumor | No vs yes | 1.00 | 0.56-1.77 | 0.996 | 0.95 | 0.43-2.12 | 0.898 | 0.98 | 0.44-2.19 | 0.960 |
| N-stage | cN0 vs cN1 | 0.52 | 0.13-2.10 | 0.357 | 0.55 | 0.08-3.98 | 0.551 | 0.45 | 0.06-3.30 | 0.435 |
| Lymphovascular invasion | No vs yes | 1.08 | 0.49-2.38 | 0.847 | 1.15 | 0.41-3.27 | 0.791 | 1.71 | 0.67-4.41 | 0.265 |
| CTx dose reduction | No vs yes | 0.64 | 0.37-1.10 | 0.105 | 0.71 | 0.33-1.54 | 0.404 | 1.16 | 0.49-2.74 | 0.737 |
| Elective lymph node irradiation | No vs yes | 1.38 | 0.88-2.17 | 0.163 | 0.87 | 0.44-1.75 | 0.700 | 1.19 | 0.69-2.07 | 0.530 |
| TURBT transurethral resection of a bladder tumor, CIS carcinoma in situ, CT chemotherapy, OS overall survival, DSS disease specific survival, LDFS locoregional disease free survival | | | | | | | | | | |

Supplementary Figure 1: Bladder Intact Event Free Survival

Supplementary Figure 2: (A) Locoregional Disease Free Survival, comparing bladder only radiotherapy (BO-RT) and bladder + elective pelvic lymph node radiotherapy (B+PLN-RT); (B) Disease Specific survival, comparing T2 vs T3-4; (C) Overall Survival, comparing T2 vs T3-4; (D) Overall Survival, compared by chemotherapy radiosensitizer; (E) Overall Survival comparing radiotherapeutic dosage
